# Disentangling the feedback loops driving spatial patterning in microbial communities

**DOI:** 10.1038/s41522-025-00666-1

**Published:** 2025-02-20

**Authors:** Alyssa Henderson, Alessia Del Panta, Olga T. Schubert, Sara Mitri, Simon van Vliet

**Affiliations:** 1 Department of Environmental Systems Science, ETH Zurich, Zurich, Switzerland; 2 Department of Environmental Microbiology, Eawag: Swiss Federal Institute of Aquatic Science and Technology, Dübendorf, Switzerland; 3 Department of Fundamental Microbiology, University of Lausanne, Lausanne, Switzerland; 4 Biozentrum, University of Basel, Basel, Switzerland

**Keywords:** Microbial ecology, Biofilms

## Abstract

The properties of multispecies biofilms are determined by how species are arranged in space. How these patterns emerge is a complex and largely unsolved problem. Here, we synthesize the known factors affecting pattern formation, identify the interdependencies and feedback loops coupling them, and discuss approaches to disentangle their effects. Finally, we propose an interdisciplinary research program that could create a predictive understanding of pattern formation in microbial communities.

## Introduction

Our understanding of microbes is largely based on experiments done in well-mixed liquid cultures, which are relatively easy to control and have high repeatability. However, suspending cells and mixing them in liquid removes an important part of the reality that most microbes face in their natural environments: a surface to stick to and grow on, a restriction in movement due to crowding in densely packed populations, and the ability to substantially alter the local physical and chemical environment through the uptake and release of molecules^1–7^.

**Fig. 1:**
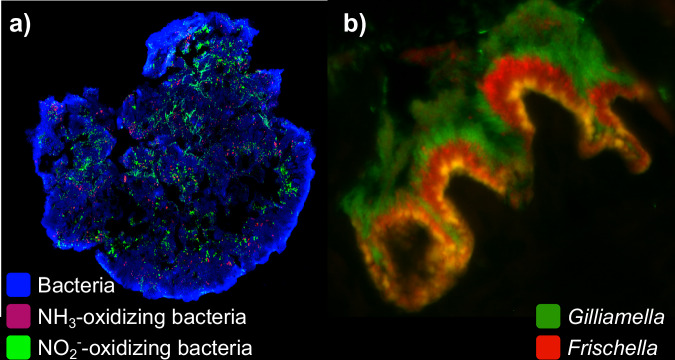
Spatially organized microbial communities in nature. **a** Bacteria (blue) in a granule from a wastewater treatment plant visualized by fluorescence in situ hybridization (FISH). Ammonia-oxidizing bacteria (red) and nitrite-oxidizing bacteria (green) exhibit spatial patterning. **b** In the pylorus region of the honeybee gut, *Gilliamella* (green) and *Frischella* (red) grow in overlapping but spatially distinct niches, here shown by FISH imaging. *Images generously provided by a) Nicolas Derlon (Eawag) and b) Philipp Engel (University of Lausanne)*.

A community’s spatial structure can either be externally imposed through the environment^8,9^ or emerge through the self-organization of cells^10–12^. In many natural environments, the availability of water, nutrients, or space limits growth to distinct locations, e.g., to pores in the soil, or to liquid droplets on surfaces. As a result, microbial communities become structured into metapopulations of spatially segregated, but potentially coupled, sub-populations^13^. However, spatial structure can also emerge in the absence of external—abiotic—spatial structure (e.g., see examples in Fig. 1)^14–19^. In these cases, spatial structure emerges as a consequence of how cell grow, move, and arrange themselves in space. Although both forms of spatial structure have important ecological and evolutionary consequences, here we focus on spatial structure formed through emergent self-organization.

Self-organized spatial patterns emerge from the growth and movement of individual cells. A cell’s growth and movement are determined by its local biological, chemical, and physical microenvironment. Cells, in turn, also shape their local environment. A community’s spatial arrangement is thus a complex property that emerges from the feedback loops between cells and their environment. This spatial arrangement is an essential property that affects the dynamics, function and evolution of microbial communities^20–25^. Understanding the ecology and evolution of microbial communities therefore requires insights into how spatial patterns arise in structured cell populations.

**Fig. 2:**
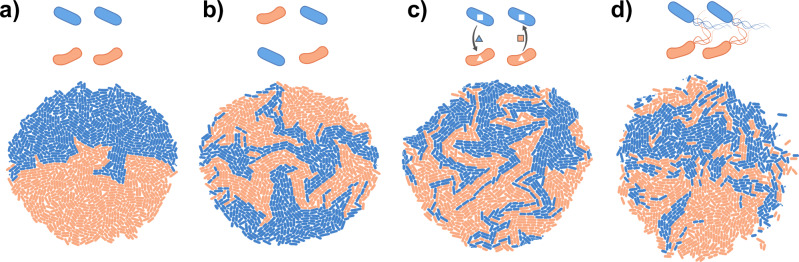
Similar spatial patterns can arise from different processes. Two-dimensional colonies consisting of two species were simulated using the gro toolbox^154^. Each colony starts from two cells of each species, which have the same (basal) growth rate. **a** No intermixing is observed when the species start from a segregated arrangement. **b** Intermixing is observed when species start from a mixed arrangement. **c** Intermixing is also observed when species promote each other’s growth in a mutualistic interaction. **d** Intermixing is also observed when species are motile, and move as they grow. While the precise patterns in b-d are not identical, each displays intermixing, and it can therefore be challenging to identify the drivers of this pattern. The parameters used in the simulations are listed in Supplementary Note 1.

While many studies have identified factors affecting pattern formation, we still lack a general predictive understanding of how the properties of cells and their environment affect spatial patterning in multispecies communities. Progress has been slow for several reasons. Firstly, many studies have used synthetic communities consisting of two nearly isogenic strains with engineered interactions^26–30^. It is still unclear if and how these findings generalize to communities consisting of multiple species that differ in their physical and biological properties. A number of studies have started using natural multispecies communities^31–36^, recently reviewed in^2–4,6,7,37^, but the increased complexity of these systems has made it challenging to derive quantitative predictions for how the properties of cells and/or their environment determine the observed patterns^38^. Secondly, pattern formation is shaped by a diverse set of properties of cells and their environments including cell shape, inter-species interactions, initial cell density, or fluid flow^38–41^. Many of these have been studied in detail in isolation; however, they are often interdependent. This complicates the interpretation of experiments and should ideally be taken into account already during experimental design. Moreover, the relationship between the properties of cells, their interactions, and the resulting spatial patterns is complex: Different mechanisms can give rise to highly similar patterns (Fig. 2), while subtle changes in the environment can significantly change the resulting patterns^42^. Identifying which parameters and processes underlie an observed pattern is thus not obvious, and how the unique properties of cells and their environment drive pattern formation in microbial communities remains an important unsolved question.

In this review, we analyze the biological, chemical, and physical factors that drive the spatial patterning of microbial communities and highlight the interdependencies, in the form of feedback loops, that connect them. We describe the experimental setups commonly used to study pattern formation and emphasize the relevance of different factors in each setup. Finally, we propose a set of complementary modeling frameworks that can be used to describe and predict observed patterns. For simplicity, we primarily focus on two-species communities in lab-based setups. However, most of the concepts discussed here can be extended to more complex natural systems, and our overall aim is to lay the foundation of a framework to identify general principles governing pattern formation in microbial communities. By synthesizing existing research and concepts, we highlight the considerations that should be taken into account when studying spatial patterns and emphasize the value of integrating knowledge across the fields of microbial ecology and biophysics.

## What generates spatial patterns?

A spatial pattern is given by the position in space of each cell type in a microbial community. These patterns can be described using several summary statistics, for example by calculating the community composition or the degree of intermixing of different species (recently reviewed in ref. ^43^). Many different metrics have been developed, and we give a non-exhaustive overview in Table 1^18,40,44–49^. Importantly, these summary statistics depend on the scale at which the system is described^46^: cells of different species might appear to mix at a certain scale, but at small enough scales, they are often surrounded by cells of their own type.

**Table 1:** Metrics to quantify spatial patterns

Metric	Description	Example
Community composition	Quantifies the number and type of all cells, ignoring their spatial locations.	• Absolute abundances • Relative abundances
Assortment or segregation	Quantifies the relative spatial locations of cells of the same or of different types. The observed distribution of distances is compared to the expected one if cells were positioned randomly. Assortment refers to cells of two types being closer to each other than expected, while segregation refers to cells of two types being further away than expected.	• Ripley’s K-function^44^ • Assortment value^18^ • Segregation index^40,45,46^ • Fourier decomposition of colony spatial pattern^47^
Sector size	Quantifies the average size of sectors of a single species in expanding colonies.	• Sector width^27^
Cluster size	Quantifies the average size of sectors of a single species in mixed-species biofilms.	• Spatial correlation length^161,162^
Intermixing	Quantifies the frequency of boundaries between clonal clusters or sectors of different cell types.	• Intermixing index^48^ • Frequency space analysis^47^ • Boundaries over area^85^
Boundary shape	Quantifies the shape of the boundaries between clonal clusters or sectors.	• Fractal dimension^152,163^ • Contour fluctuations^49^

This table gives a non-exhaustive overview of common metrics used to quantify spatial patterns (see ref. ^43^ for additional metrics). Different metrics are often correlated with one another, e.g., for a given community composition, more intermixing implies smaller sector sizes and lower segregation scores. Moreover, many metrics are scale-dependent, e.g., segregation scores depend on the length scale at which the pattern is quantified.

**Fig. 3:**
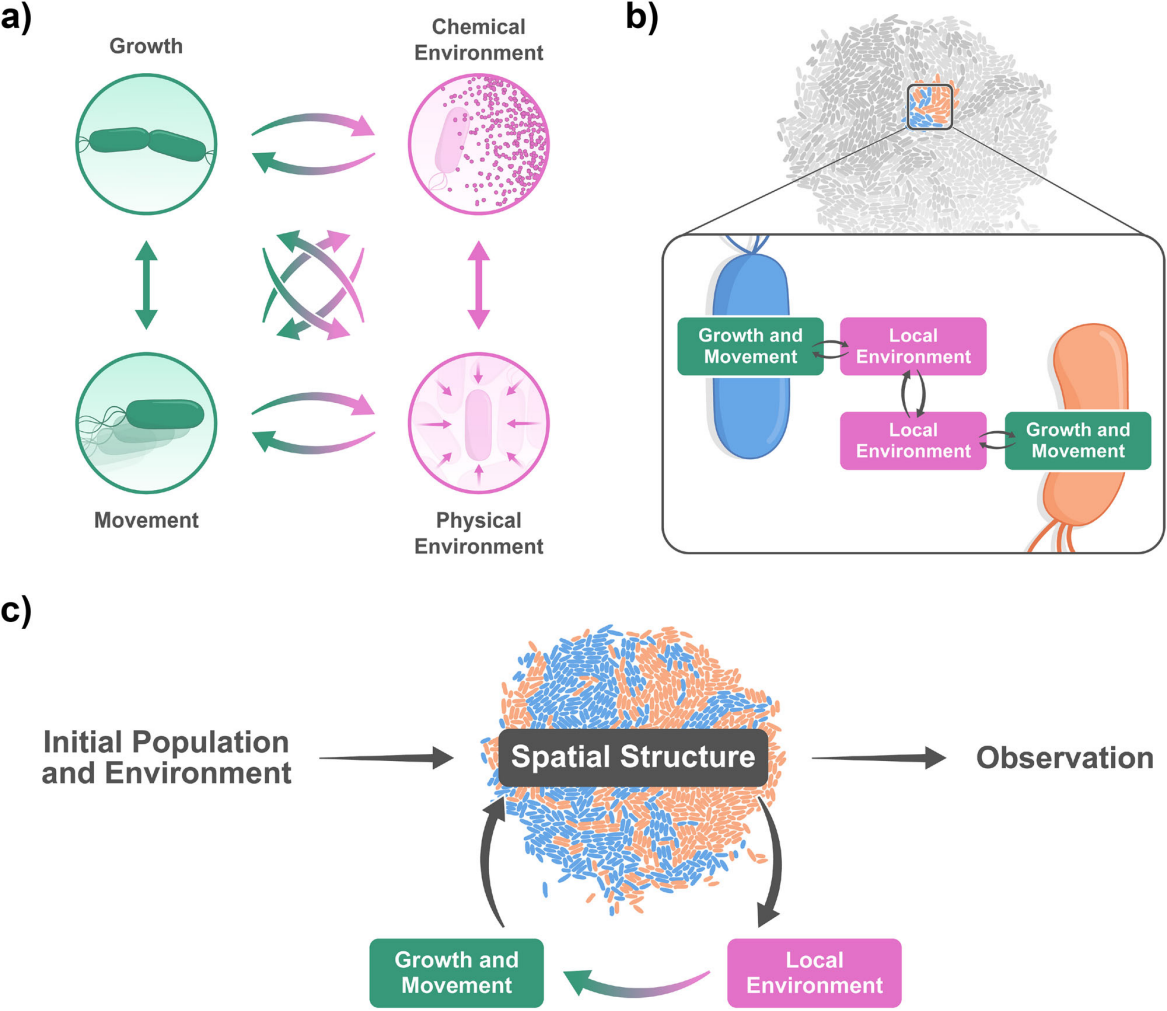
Factors affecting pattern formation, and their interdependencies. **a** The growth and movement of a cell is determined by properties of the chemical and physical environment, which are in turn influenced by the cell’s growth and movement. This leads to feedback loops between cells and their environments. **b** The local environmental changes caused by a cell’s activity can be transmitted to the local environment of neighboring cells, leading to cell-cell interactions. **c** These processes cause an initial population to grow and move according to a changing environment, leading to the emergence of spatial patterns. Visual design of the figure was improved by Joana Carvalho.

Pattern formation in microbial communities depends on a high-dimensional parameter space. The factors affecting spatial patterning can be broadly grouped into three categories: properties of individual cells, properties of their local environment, and properties of the feedback loops through which cells and environments affect one another (Table 2 and Fig. 3a).

**Table 2:** Definitions of factors and feedback loops

Initial Conditions	
Initial population	The identity, location, and state of all cells present at the start of an experiment or simulation.
Initial chemical environment	The concentration profiles of all chemicals initially present in the environment at the start of an experiment or simulation.
Initial physical environment	The initial state of the physical environment, including boundary conditions, flow profiles, etc. at the start of an experiment or simulation.
**Primary factors**	
Growth	The rate at which cells divide and form new biomass.
Movement	The rate and direction in which cells move either due to active movement or passive movement in response to external forces.
Chemical environment	The type, concentration, and distribution of all chemicals that are present in a cell’s environment as well as their fluxes. It includes the concentrations of nutrients, toxins, signaling molecules, matrix components, and surfactants.
Physical environment	The set of all physical factors that exert a force or physical constraint on cells or that otherwise affect cell growth and movement. It includes the thermodynamic state (e.g., temperature), geometry (e.g., dimensionality, boundaries), surface properties (e.g., viscosity, roughness), and hydrodynamic properties (e.g., water activity and flow rate) of the environment. Cells themselves are part of the physical environment as well, because cell crowding can lead to significant friction and pressure.
**Coupling and feedback loops between factors**	
Coupling between chemical and physical environment	A single factor (e.g., surfactant production) can simultaneously affect the chemical and physical environment.
Coupling between growth and movement	Cell growth often leads to movement (e.g., as cells increase in volume they push around their neighbors), while movement affects cell growth by moving a cell to a different local environment (e.g., moving to an area with more favorable environmental conditions).
Chemical environment-growth feedback	The chemical environment influences cell growth by determining a cell’s metabolic activity and growth. In turn, cellular metabolism changes the chemical environment, as growth can cause depletion of nutrients and accumulation of byproducts.
Physical environment-movement feedback	The physical environment influences cell movement by imposing forces and constraints (e.g., cell movement is constrained by cell crowding or boundaries of a growth chamber). In turn, cell movement changes the physical environment (e.g., movement to a new region increases cell density and crowding in that region)
Chemical environment-movement feedback	The chemical environment influences cell movement through chemotaxis. In turn, movement can change chemical gradients, as a cell can move to a new area and secrete chemicals locally.
Physical environment-growth feedback	The physical environment influences cell growth directly through temperature and pressure. Physical features such as flow can also influence chemical distribution, thereby influencing growth indirectly. In turn, cell growth contributes to physical effects, such as pressure from cell crowding.
**Emergent interactions**	
Metabolic interactions	Metabolic interactions emerge as neighboring cells become coupled through their shared chemical microenvironment.
Physical interactions	Physical interactions emerge as neighboring cells become coupled through their shared physical microenvironment.

Here we give detailed definitions of all important factors and feedback loops that need to be considered to understand, model, and predict pattern formation.

### Cells

Spatial patterns emerge from the positions of individual cells in space. These positions are dynamic: Over time, cells grow and move, and, as a result, spatial patterns can change. Three factors related to individual cells are therefore relevant to pattern formation: initial positions, growth and death, and movement.

First, as also illustrated in Fig. 2, the initial positions of cells relative to one another can affect pattern formation^40^. In many experiments, such as colony range expansions, variation in the initial ratios of cell types, cell densities, and cell positioning can generate different patterns^30,50–52^. While it is known that the initial configuration of cells is relevant to pattern formation, systematically studying the relationships between initial positions and spatial structure in the lab is challenging because it is difficult to experimentally control the precise positions of different species (but see ref. ^45^). Stochasticity therefore often influences the initial configuration of cells in experiments, which can subsequently shape pattern formation. However, certain statistical properties of spatial patterns, such as their composition, or degree of assortment, can be robust to these stochastic fluctuations^27,28^.

The growth and death rates of cells also shape spatial patterns. The growth rate of cells relative to that of their neighbors determines which cell type will occupy previously empty positions or push neighboring cells away^53^. Death rates can also influence patterns by freeing up space^18,54^ or by changing where growth happens^55^. The growth and death rates of cells are primarily determined by how cells respond to their local chemical^56^ and physical environment^57–60^, which will be discussed in subsequent sections. However, they can also be modified through direct contact-dependent interactions with neighboring cells^61^. Moreover, cell death due to phages and predation can also change spatial patterns^18,54,55^.

Finally, active and passive movement can affect pattern formation^38,50,62,63^. Active motility occurs when microbes use energy to move in their environments using appendages, such as flagella and pili^64–66^. This movement can be undirected or directed by environmental stimuli (e.g., chemotaxis)^66,67^. Passive movement occurs in response to an external physical force, such as fluid flow^68^, which can affect the initial distribution of cells on a surface and thereby influence the degree of segregation^69^. Moreover, as microbes grow and crowd their local environments, cells can also move as they push against one another^70^. Both active and passive movement are constrained by the geometry of a cell’s environment^71^ and are affected by a cell’s physical properties, such as it size, shape, and adhesiveness^72^. For example, differences in cell shape or size can lead to spontaneous sorting of cells^39^.

### Local physical and chemical environment

Physical and chemical environmental properties influence pattern formation by affecting the growth and movement of cells (Fig. 3b). In spatially structured systems, environmental properties are often heterogeneous even at the micrometer scale, with each cell experiencing a different local microenvironment^73^.

A cell’s physical environment is the set of all properties that affect cellular growth and movement through physical mechanisms, such as temperature, surface properties, and hydration levels (see Table 2)^74–76^. The physical environment strongly affects cell growth and movement through the effects of physical forces (recently reviewed in refs. ^57^^,^^58^). These forces can be exerted by physical constraints of the setup in which cells are embedded, the media in which they grow, or by other cells^30,77,78^. For example, physical constraints that limit the space available for cells to grow, and friction with the boundaries of this space, can influence the extent to which cells tend to stay trapped in the environment^30^. The environment can also exert different forces on cells depending on their viscosity. A more viscous environment can limit cell movement and promote segregation of cell types^78^. Cells themselves modify the physical environment. For example, cells may produce extracellular matrix, which can regulate osmotic pressure and promote expansion of the community^79^. Additionally, by growing and occupying space, cells contribute to local crowding, which exerts pressure on other cells^57^. This crowding can lead to collective cell motion^70^, affect competition outcomes^53^, or even lead to growth arrest^80^. The effect of these forces depends on cell properties such as size, shape, and rigidity^30,39,81^.

A cells’s chemical environment is defined by the concentration and fluxes of metabolites, toxins, and other chemicals. It affects growth by determining the rates at which cells accumulate biomass and divide^56,82^. The chemical environment can also influence cell movement through chemotaxis along chemical gradients or through a general upregulation of motility^66,83^. The overall nutrient availability is also an important factor, as it determines the final density that is reached before growth ceases. Chemical features cannot always be disentangled from physical effects. For instance, chemicals such as surfactants and exopolysaccharides change the physical environment by affecting surface tension and viscosity^78^. Physical properties like fluid flow can also modify the distribution of molecules in space, altering the chemical landscape^84^. Moreover, higher crowding of cells leads to higher uptake of metabolites and increases the heterogeneity of the chemical environment^23,73,85–87^.

### Feedback loops between cells and their environments

While environmental properties affect cellular growth and movement, cells, in turn, influence the environment (Fig. 3b). The physical environment is, for example, affected by cell crowding^81,88^, while the chemical environment is altered through the depletion of nutrients, and secretion or leakage of metabolites^20,23,89,90^. Moreover, the effect of a cell’s environment on its movement and growth depends on the cell’s metabolic capabilities, motility machinery, cell shape, surface properties, and many other cellular properties^58,91^. There are thus many interdependencies and feedback loops between cellular and environmental properties.

The tight feedback loops between cells and their environment have two key implications for spatial patterns. First, the factors affecting spatial patterning cannot be treated independently. Because growth, movement, and environmental properties are intertwined, it is challenging to isolate the effect of a single factor on spatial structure from experimental data. For example, changing the flow rate in a microfluidic setup changes both the physical forces exerted on the microbes while also increasing the rates at which nutrients are introduced and metabolic byproducts removed. If the change in flow rate leads to a change in the spatial structure, it is thus challenging to pin down its causes. Careful quantification of these different effects, and their interactions, is therefore crucial to gaining mechanistic insights into pattern formation^92,93^.

Second, because nearby cells share the same micro-environment, these feedback loops give rise to emergent cell-cell interactions (recently reviewed in refs. ^14,16^ for metabolic and refs. ^58,94^ for physical interactions). When a cell modifies its local chemical or physical environment, this change can affect neighboring cells and cause them to grow or move differently^85,86,89,95–98^, which in turn influences spatial structure^26,52,90,99–101^. For example, a given microbial cell can “cross-feed" others by secreting metabolites that change the chemical environment^102–104^, or it can cause crowding by moving to a new position, both of which influence the growth and movement of neighboring cells^53,80,105,106^.

### The challenges of interpreting spatial patterns

**Fig. 4:**
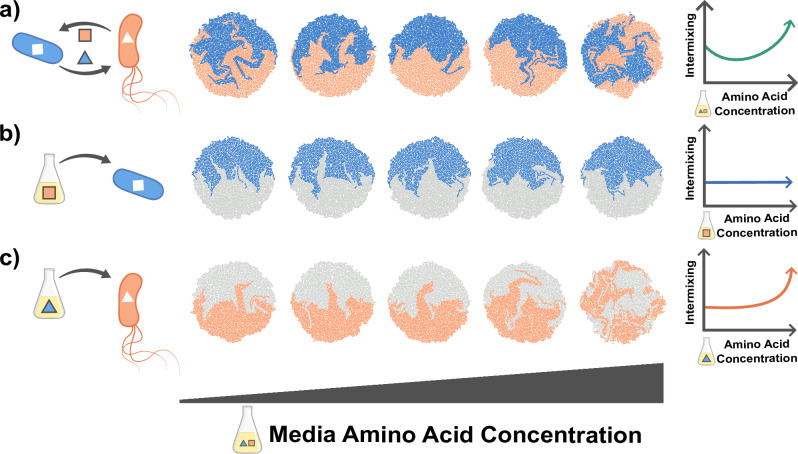
Importance of disentangling factors driving pattern formation. Pattern formation of two cross-feeding amino acid auxotrophic strains, simulated using the gro toolbox^154^. One of the two strains is non-motile (blue), while the other is motile (orange) with motility increasing with amino acid concentration. The interaction strength is tuned by setting the amino acid concentrations in the media, with higher concentrations reducing the metabolic dependency between the two strains. In the simulations, each microcolony is initialized by two cells from each type. **a** The amino acid concentration in the media influences intermixing non-monotonically. To better understand the pattern formation in this mixed colony, we consider the self-patterns formed by each strain individually in varying media compositions. These can be visualized using differential color labeling where half the founder cells of each species are colored in gray. **b** Amino acid concentrations do not substantially affect the patterns for the blue species. **c** Amino acid concentrations increase intermixing in the orange species as its motility increases at higher amino acid concentrations. The parameters used in the simulations are listed in Supplementary Note 1.

In this section, we will use a simple conceptual model to illustrate how the interdependencies discussed above can make it challenging to create a predictive understanding of how properties of cells and their environments affect spatial patterning. Let us consider pattern formation in colonies of two auxotrophs, e.g., two strains that each cannot produce an essential amino acid, but that can complement each other’s growth by secreting these amino acids into the environment. The strength of the dependency can be tuned by changing the concentration of amino acids in the externally supplied growth medium. As the external amino acid concentration is increased, the dependency weakens, and we expect the degree of intermixing to decrease. However, in a scenario where one strain exhibits increasing motility as amino acid concentration increases, we find that while intermixing initially decreases, at higher external amino acid concentrations it increases again (Fig. 4a)^107^. To understand this phenomenon, we need to consider how amino acid concentrations affect each of the species in isolation: pattern formation in the first strain is not affected by increasing amino acid concentrations (Fig. 4b). However for the second species, we see increased intermixing (Fig. 4c). These two observations, coupled with our knowledge of the interaction between the strains, allows us to understand the spatial pattern that develops when they are grown together.

These simulations illustrate that it is essential to identify all factors that change as a result of an experimental manipulation. In our example, the observed change in spatial patterns could only be explained by also quantifying how changing the chemical environment affected cell motility (Fig. 4). Importantly, it is often not clear a priori which factors might be affected by an experimental manipulation. In the current example, the unexpected non-monotonic relation between cell mixing and interaction strength would probably have alerted the experimentalist that there was an unidentified confounding factor at play (Fig. 4a), leading to additional control experiments (Fig. 4b, c). However, in many cases the effects of the confounding factors are likely more subtle and could easily be missed, leading to potentially erroneous interpretations of the experimental results. It is therefore essential to always be aware of the potential presence of hidden confounding factors and plan control experiments accordingly.

In the remainder of the article, we will discuss how we can create a quantitative understanding of these feedback loops between cells and their environment using experimental and computational approaches.

## Experimental setups to study spatial patterns

**Fig. 5:**
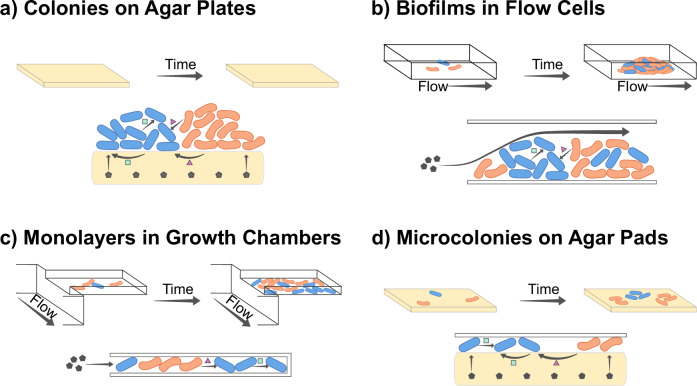
Overview of experimental setups to study spatial patterns. Experimental setups differ in multiple ways, including the space accessible for cell growth, the way that nutrients diffuse, and how interactions play out. For each experimental setup, the top panel represents a basic overview, while the lower panel displays how chemicals travel within the system. Gray hexagons represent nutrients in the media, while pink triangles and green squares represent chemicals released by the cells. Schematics are shown for: **a** Colony biofilms on agar plates. **b** Biofilms in microfluidic flow cells. **c** Monolayers in microfluidic growth chambers. **d** Microcolonies on agar pads.

In the previous sections, we have argued that the environment is an important determinant of the processes that shape the spatial structure of microbial communities. As a result, the same cell types growing in different environments can exhibit distinct spatial patterns. For example, whether cells can grow in two or three dimensions, the shape of physical boundaries, or the spatial and temporal dynamics of nutrient supply can all change emerging patterns qualitatively or quantitatively. In the following, we analyze some of the most commonly used experimental setups with this perspective in mind and discuss how spatial patterns are influenced by the properties of each setup (Fig. 5, Table 3).

**Table 3:** Comparison of common experimental systems

	Colonies on agar plates	Biofilms in flow cells	Monolayers in growth chambers	Microcolonies on agar pads
**Scale**				
Cell number^a^	>10^9^	10^3^–10^6^	100–1000	10–500
Length scale	1–10 mm	10–500 μm	10–100 μm	5–20 μm
Imaging resolution	Population-level	Single-cell for small biofilms	Single-cell	Single-cell
**Physical environment**				
Growth interface	Agar-air	Liquid-glass	Agar-liquid-glass	PDMS-liquid-glass
Density	High	High	High	Low
Physical constraints	None	None	Restricted in all dimensions	Restricted in z-dimension
Dominant forces	Crowding, friction, surface tension	Crowding, shear force	Crowding, friction	Crowding, friction, surface tension
**Chemical environment**				
Nutrient supply	From agar, finite resources	From liquid, replenished	From liquid, replenished	From agar, finite resources
Dominant gradients	From air & agar interface to biofilm core	From liquid interface to biofilm core	From opening to death end of chamber	From edge to center of microcolony
Steady-state gradients?	Quasi-steady state^b^	Quasi-steady state^b^	Yes	No
Diffusion within community	Through crowded biofilm & agar	Through crowded biofilm	Through crowded chamber	Through agar
Chemical coupling between communities?	No	Yes, through flow and diffusion	No	Yes, through diffusion
Movement of cells between communities?	No	Yes, through flow and motility	No	Yes, through surface motility

^a^Cell numbers depend on setup, species, and growth media, a typical estimate is shown.

^b^Quasi-steady state gradients are formed in large colonies and biofilms, where the chemical concentrations as a function of the distance from the edge are approximately constant in time.

### Colonies on agar plates

Colony biofilms are macro-scale (mm to cm) communities that grow on a nutrient-containing surface (recently reviewed in refs. ^108,109^). In a typical experiment, a mixture of cell types is placed in the center of an agar plate and cells grow outwards on the agar–air interface. Expanding colonies experience no physical boundaries, but rather, after an initial exponential growth phase, expand at a constant velocity^110,111^. The radial growth rate is determined by physical forces at the colony edge, while vertical growth is limited by the depletion of substrates^112,113^. The actively growing population is thus restricted to the colony edge, where lineages compete to occupy empty space. Thus, faster-growing lineages are more likely to form sectors in the growing colony. However, even if all lineages grow at equal rates, sectors form due to genetic drift^49,111^. Interactions between cell types also modulate the spatial patterns, for example, metabolic dependencies typically lead to small sectors with more intermixing^27,28^. While colony biofilms are primarily used to study isolated biofilms, these assays can also be used to study interactions between biofilms^114^.

Colony assays lack single-cell resolution and are more commonly used to study population-level behavior. Lineage growth rates can be quantified by destructively sampling the colonies and comparing initial versus final lineage frequencies by counting colony-forming units^61^. However, this cannot capture the time and spatial dependence of interactions and pools together the effects of metabolic and physical interactions. Imaging can also provide information about the interaction between cell types. For example, the sectors of the fittest strain tend to increase in width throughout expansion^49,111^ and the degree of intermixing increases when metabolic metabolic dependencies are stronger^27,28^. Many other factors, such as cell shape^39,49^, physical interactions^115^, killing interactions^54^, or even lag times^52^ affect the shape of sectors. While it is generally hard to obtain single-cell growth rates and movement, adaptive microscopy techniques do allow for the tracking of individual cells at the colony edge^116^. Moreover, recently, additional methods have been developed to study the transcriptional and metabolic activity of cells for distinct spatially resolved subpopulations^89^.

### Biofilms in microfluidic flow cells

In microfluidic devices, cells grow attached to a surface, to each other, or are physically trapped in growth chambers, while nutrients are provided via the liquid media in which they are immersed (recently reviewed in ref. ^117^). These devices can be designed either as open systems where chemicals flow into and out of the system continuously, or as closed batch systems where cells and chemicals are inoculated and the device is sealed.

The simplest microfluidic setups consist of a rectangular flow channel in which biofilms grow attached to the glass surface. With time-lapse microscopy the growth and movement of cells can be followed through the entire biofilm life cycle: from initial surface attachment and microcolony formation, to the growth and maturation of three-dimensional biofilms, to the eventual dispersal of the biofilm^118^. Recent advances in image analysis techniques allow for the analysis of spatial patterns at single-cell resolution in biofilms of up to a few thousand cells^119,120^. Moreover, transcriptomic profiles can be obtained for distinct subpopulations^89,121^.

Flow cell biofilms differ from colony biofilms in a few important ways. First, the chemical environment is qualitatively different. In flow cells, both substrates and oxygen enter from the biofilm–liquid interface, whereas in center of colonies they enter from opposite sides (at the colony edge gradients are more complicated). Second, different physical forces are relevant, with shear forces dominating in flow cells and friction dominating in colony biofilms. Finally, whereas colony biofilms are mostly grown in isolation, flow cells often contain many separate biofilms that are coupled through the exchange of cells and chemicals. Upstream biofilms can significantly alter the chemical environment of downstream biofilms^84^. Through passive and active motility, cells can migrate between biofilms, thereby allowing for major changes in spatial arrangement even in mature biofilms^122^.

### Monolayers in microfluidic growth chambers

Microfluidic growth chambers can be used to physically constrain cells to grow in mono-layers, facilitating quantification of growth rates and gene expression at the single-cell level^123^. These devices typically consist of a flow channel, with small growth chambers branching off to the sides, creating many independent compartments that are shielded from the fluid flow^90,124,125^. The high density of cells and limited flow can create micro-scale chemical gradients^23,123,126,127^ and short-range cell-cell interactions^85,86^ that are similar to those in three-dimensional biofilms.

By constraining growth to two dimensions, it is substantially easier to track the growth and movement of cells in microfluidic growth chambers than in three-dimensional biofilms. These chambers are therefore well suited to study in detail how the location of a cell relative to the nutrient source, or to other cells, affects its growth and activity. However, the geometric constraints of the chambers assert strong physical forces, that can affect cell physiology and pattern formation^71,124,128,129^. This can make it challenging to study cell types that differ strongly in their size or growth rates. For example, chambers quickly become dominated by the fastest-growing cell type^30^. However, this can be avoided using clever designs: microfluidic chips with an undulating bottom trap cells and can allow for more stably coexisting lineages within the same chamber^129^.

### Microcolonies on agar pads

In agar pad experiments, very low-density cultures are added to a piece of agarose gel and covered with a glass coverslip. The initial location of cells is typically random, however it can be controlled by printing initial patterns^45^. These cells then grow into microcolonies of up to a few hundred cells. Like in microfluidic growth chambers, cells are primarily constrained to grow in a mono-layer, allowing for single-cell resolution imaging using conventional microscopes. However, unlike growth chambers, cells are completely free to move in the other two dimensions. Agar pads can thus be used to study active movement toward a cue over longer distances, such as toward a chemical signal secreted by another cell type in a different microcolony^62,66^.

While microfluidic approaches require specialized equipment and are challenging to set up, agar pads are relatively simple to use, making them ideal for high-throughput screening of interactions between species^130,131^. However, their chemical and physical environment is qualitatively different from those in the other setups described so far. Because all cells are directly connected to the surrounding medium (i.e. the agar pad), gradients in nutrients and excreted molecules are weaker (Fig. 5). The effect of interactions can thus be qualitatively different from those found in dense biofilms or growth chambers^132^. Moreover, due to the small distances between microcolonies, cells interact simultaneously within^133,134^ and between microcolonies^66,130,131,135^. This can make it challenging to quantify the strength of interspecies interactions. However, with careful data analysis and control experiments, quantification of interactions is still possible^131^. Finally, due the short distances between microcolonies, motility plays a much bigger role than in large colony biofilms^66^.

### Choosing an experimental setup

The setups described above all have their own strengths and weaknesses, and the choice of which to use depends on the question at hand (Table 3). Care must be taken to consider all confounding factors that can affect the process of interest and choose the setup that minimizes these factors. Moreover, the choice also depends on the natural system of interest. For example, agar pads can be used to model the competition between neighboring microcolonies that occurs in environments such as the soil^8,136^, while flow cells model the environment experienced by biofilms in aquatic environments^137,138^.

In addition to the setups described above, many more experimental models have been developed to mimic specific natural systems. For example, microfluidic devices have been developed to study diffusion-mediated interactions^139^, the colonization of structured habitats^140,141^ and free-floating marine snow particles^142,143^, or the dynamics of filamentous biofilms (streamers)^92,93^. Other groups have developed systems to study nonsurface attached biofilms^144^, recently reviewed in ref. ^68^. Finally, organ-on-a-chip^145,146^ and plant-on-a-chip^147^ devices could be used to study pattern formation in the context of host environments.

## Building predictive models

**Fig. 6:**
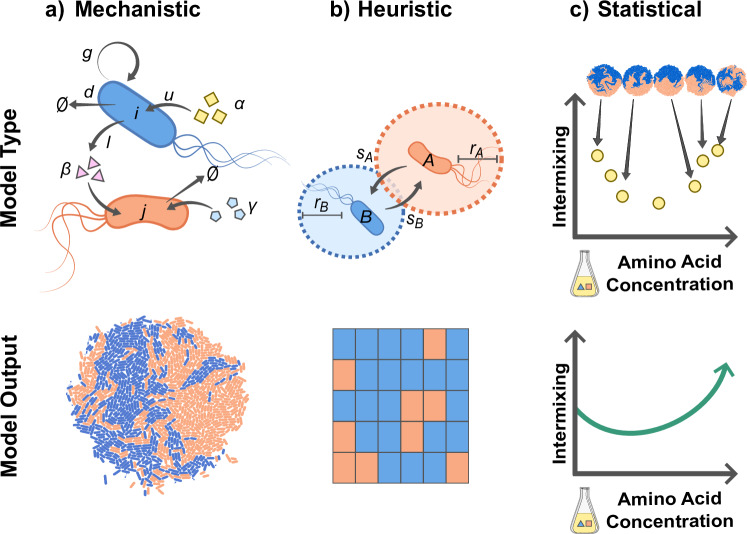
Overview of modeling frameworks to study spatially structured microbial communities. **a** Mechanistic models describe metabolic and physical interactions explicitly and thus require information such as the uptake (*u*) and leakage (*l*) rates of chemicals (*R*_*α*_, *R*_*β*_, and *R*_*γ*_), as well as how these chemicals influence the growth (*g*) of cells (*i* and *j*). Mechanistic information can for example be used to parameterize an agent-based model where cells in a colony grow in response to chemicals that are represented by explicit reaction-diffusion equations. **b** Heuristic models describe interactions more coarsely, using parameters such as the interaction range (*r*) and strength (*s*), with strength describing the effect of the interaction on growth. These values can parameterize cellular automata, where cells are placed on a two-dimensional grid and the dynamics is described by a death-birth update rule. **c** Statistical models do not rely on mechanistic parameters, but rather aim to fit a function that predicts the value of one or more variables based on the value of other variables. For example, a model could be fit that relates an independent variable such as external amino acid concentration to a dependent variable, such as intermixing of strains.

Mathematical and computational models can be helpful either to create a conceptual understanding of pattern formation in general, or to describe or predict it for a specific experimental system (e.g., a specific microbial community in a specific experimental setup). In the following, we will discuss three different predictive modeling frameworks to study spatial pattern formation that are more or less appropriate depending on the level of understanding and quantitative characterization of the experimental system (Fig. 6).

### Mechanistic models

The goal of mechanistic models is to explain and predict pattern formation through biological, chemical, and physical interactions. This requires the formulation of a mathematical model with explicit metabolic and physical interactions^64,99,148–155^. Mechanistic models keep track of the positions and orientations of individual cells (agent-based models), as well as the concentrations of exchanged metabolites. The concentrations of metabolites are obtained from reaction-diffusion equations describing the uptake and externalization of metabolites by cells, and diffusion in the medium. Cell biomass increases through the uptake of metabolites. Chemical interactions are thus modeled mechanistically by considering explicitly how cells change their local environment through the uptake and externalization of chemicals, how this in turn affects neighboring environments through diffusion, and finally how this affects the growth of these neighboring cells. After the biomass has reached a certain threshold, cells divide and new-born cells are assigned a new position and orientation. Physical interactions between cells can be modeled in several ways, for example by preventing two cells to occupy the same position. Recent work has extended this approach by implementing agent-based genome-scale models, which include a detailed description of each individual cell’s metabolic activities^88^. Depending on the experimental setup and the question of interest, some simplifications can be made. For example, if cell shape and motility can be ignored, the agent-based model can be simplified to a cellular automaton^53,101,156^. To make quantitative predictions for a specific experimental system, uptake and leakage rates as well as diffusion constants for all exchanged compounds must be measured. Moreover, all physical and biochemical parameters governing the growth and movement of cells need to be defined. In practice, this can be challenging. However, many of these parameters can be estimated from batch experiments in liquid media or by fitting single-cell measurements obtained from experiments with one of the setups described above^43^.

### Heuristic models

In heuristic models, the variables of interest are not biochemical parameters but coarse-grained variables that describe the main interaction attributes. Instead of explicitly modeling how chemical interactions arise from the exchange of chemicals, the effects of these interactions are summarized by considering how the growth of a focal cell depends on the composition of its local neighborhood. Interactions can thus be described by their range (i.e., the size of the local neighborhood) and the growth function that maps the composition of this neighborhood to the focal cell’s growth rate. These coarse-grained variables can either be directly estimated from experimental data, or derived from fundamental biochemical parameters using a biophysical model. The goal of heuristic models is to explore trends of pattern formation that can be generalized beyond the mechanistic details of any specific experimental system, while maintaining a connection to the underlying mechanisms. Heuristic models can be developed with different levels of abstraction: for example if cells interact through the exchange of multiple different chemicals, heuristic models can either include all of these interactions independently, or combine them into a single effective interaction^157^. Likewise, the growth of cells can be simulated by keeping track of how cells increase in biomass and divide once they reach a certain threshold, however these details can also be abstracted away by assuming simple death-birth dynamics where random cells are removed from the grid and replaced by their fastest growing neighbor. This last approach has been used successfully to create a graph-based model of pattern formation in a consortium of *Escherichia coli* auxotrophs growing in microfluidic growth chambers, and allowed for the prediction of community composition and structure from the interaction strength and ranges^101^.

Heuristic approaches can also be used for colony experiments via reaction-diffusion equations which consider continuum cell densities^111,158^. These models can predict sector properties such as fitness and can thus be used to infer interaction parameters (e.g., by fitting the data with a Lotka-Volterra model^159^).

### Statistical models

Statistical models aim to find correlations between spatial features and measured parameters, regardless of the mechanistic details. For example, regression models can be used to identify how a dependent variable (e.g., the degree of mixing between two strains) depends on an experimentally controlled environmental variable (e.g., the nutrient concentration in the medium) or property of the strains (e.g., their level of motility). Moreover, machine-learning models can be trained to infer interactions from spatial patterns^160^. Statistical models are ideal when interaction mechanisms are unknown, or when large-scale screens are performed. For example, ref. ^49^ found that the final sector width in a colony depends on the roughness of the boundaries between sectors.

### Choosing a modeling framework

While mechanistic models can make highly accurate predictions, they can be challenging to parameterize. On the other hand, statistical models require no parameterization at all, but also do not provide mechanistic insights. Heuristic models lie at an intermediate level of complexity and share the positive aspects of both: they help to identify trends with a few intuitively comprehensible parameters that can either be directly measured or calculated from the biophysical parameters. For this reason, they might be best suited for finding general rules for pattern formation in microbial communities.

## Conclusions

The spatial structure of microbial communities shapes their dynamics, function, and evolution. As such, understanding the drivers of spatial structure is of great importance. Spatial patterns arise from the growth and movement of cells, which in turn are determined by the properties of cells and those of their chemical and physical environment. These properties are tightly interconnected and are part of intricate feedback loops. It is thus challenging to isolate the effect of a single factor on spatial features and to understand how they combine to affect overall patterns.

Although the spatial structure has been intensely studied, there are still many open questions (Table 4). So far, research has primarily focused on simple biological systems and interactions have mostly been studied between closely related cell types (e.g., strains of the same species). Moreover, the effects of varying the physical and chemical properties of the environment have mostly been studied by varying a single factor at the time in a largely unsystematic way (i.e., different factors were studied using different systems complicating direct comparisons of their effects). This has yielded, at best, a partial understanding of the drivers of pattern formation. We are thus still unable to identify general principles that apply across biological and experimental systems. Consequently, predicting pattern formation in synthetic and natural microbial communities remains inaccessible.

**Table 4:** Open Questions

Question	Description
Are there general principles that govern pattern formation?	We know of many factors that affect pattern formation (Table 2), but do these affect all communities in the same way, or is the effect specific to a particular system or environment? And, can we derive general statements regarding which factors are most important under which conditions, or do we need to re-evaluate this for each system of interest?
How do different factors affect one another?	The factors that control spatial pattern formation are connected through many interdependencies and feedback loops (Table 2, Fig. 3). Changing one factor often changes others as well, but we lack a quantitative understanding of how different factors are related.
Can we use spatial patterns to infer cell-cell interactions?	Spatial patterns are shaped by cell growth and metabolic interactions but also depend on physical properties of cells and the environment as these control how cells move. What is the relative importance of these factors? If cell-cell interactions dominate, observed patterns could be used to infer the type of metabolic interactions, but if physical effects dominate spatial patterns, such inference approaches can be misleading.
Can cells arrange themselves in a way that increases their growth rate?	A cell’s location strongly affects how well it grows. But to what extent do cells control their location? Could active motility allow cells to achieve a better arrangement? Moreover, could cells evolve towards more optimal patterns if they are co-evolved over longer periods of time, or does competition between species prevent communities from reaching such optimal arrangements?
What are the relevant spatial scales in complex communities?	Two important length scales are the interaction range and the patch size, but how are these determined? For simple systems, these length scales can be calculated from biophysical models, but are these models still valid in more complex systems?
What is the role of temporal dynamics?	The physical and chemical environments change substantially over time as cells colonize new environments and grow into dense communities. Are there general principles governing the role of temporal dynamics in pattern formation?
What is the role of cell death in shaping patterns?	Cell death due to antagonistic interactions, phages, or predation, changes the physical and chemical environment by removing cells and releasing their chemical content. Death could thus affect pattern formation by determining how and where cells can grow (see recent review by ref. ^18^). Can we deepen our understanding of the role of cell death in pattern formation, and understand its interdependence with the other factors?

While remarkable progress has been made in developing the computational, experimental, and conceptual tools required to unravel spatial pattern formation in microbial communities, many open questions remain. By systematically designing experiments and simulations that consider the framework presented in this article, the answers to many of these questions may be within reach.

To fill this knowledge gap, it will be essential to systematically study how the properties of cells and their environment, as well as the feedback between them, affect pattern formation across a wide range of biological systems and environments. This requires three main lines of research. First, we must quantify how different factors are connected through feedback loops and other interdependencies (“What generates spatial patterns?”, Table 2). This requires carefully designed experiments where factors are systematically changed, alone and in combination, while using control experiments to disentangle the effects of possible confounding factors (e.g., as illustrated in Fig. 4). Moreover, bio-printing approaches could be used to experimentally manipulate spatial patterns directly, allowing for easier quantification of how these patterns affect cell growth and movement^45^. Second, we need to create an understanding of how the environment or experimental setup affects pattern formation (“Experimental setups to study spatial patterns”). This requires studying the same biological system across different experimental setups and environmental settings. Finally, to find general principles, it is essential to study a wide range of biological systems across a set of well-defined but diverse environments (e.g., using the four setups described in “Experimental setups to study spatial patterns”). Mathematical models could then be used to identify general principles by describing how different factors interact to affect overall pattern formation (“Building predictive models”). Such cross-system comparisons could be facilitated by developing standardized experimental and data reporting protocols.

By recognizing the fundamental processes that underlie pattern formation, we can design experiments and models to study the intricate relationships between biological, chemical, and physical factors that affect spatial patterning. Understanding these relationships can lay the foundations to understand and predict pattern formation in increasingly complex microbial ecosystems. These insights will ultimately be needed to effectively engineer microbial communities to improve human and planetary health.

## Supplementary information

Supplemental Information

## Data Availability

No datasets were generated or analysed during the current study.
