# Supplementary material for: Disentangling the feedback loops driving spatial patterning in microbial communities
Source: NPJ Biofilms Microbiomes. 2025 Feb 20;11:32. doi: 10.1038/s41522-025-00666-1 (PMC11842706; doi:10.1038/s41522-025-00666-1)
Supplement: Supplementary file 1 — Supplemental Information [file 41522_2025_666_MOESM1_ESM.pdf]

Supplementary Note 1

Description of simulations

The simulations in Figures 2 and 4 of the main text were performed using the symbiosis example script of the Gro software [Gutiérrez 2017]. Default values from the Gro symbiosis script were used for all parameters and functions, unless otherwise specified below.

Simulations shown in Figure 2

Parameter values

**Parameter**	**Value**
signal diffusion rate	0.5
signal degradation rate	0.1
maximum uptake rate, k_1	1
half saturation uptake constant, k_2	1
maximum growth rate, k_3	0.1
half saturation growth constant, k_4	1
time step of simulation, *dt*	.02 (default)

Initial cell positions

Panel	Coordinates of pink cells	Coordinates of blue cells
a	(0,-20),(0,-20)	(0,20),(0,20)
b	(0,-20),(0,20)	(0,-20),(0,20)
c	(0,-20),(0,-20)	(0,20),(0,20)
d	(0,-20),(0,-20)	(0,20),(0,20)

Growth equations—independent growth

For panels a, b, and d, the following equations were used to define the cellular growth rate z:

$$y = 10$$
$$n = n + dt * y$$
$$z = k_3(n/volume)/(k_4 + (n/volume))$$

Where y is the rate of import of a nutrient whose intracellular abundance is given by n. This abundance is converted to a concentration by dividing by the cell volume.

Growth equations—cross-feeding

For panel c, where crossfeeding was modeled, the following functions were used to define secretion of nutrients and the dependence of growth on the cross-fed nutrient:

$$emit\_signal\ (\ i,\ 10\ )$$
$$x\ =\ get\_signal\ (\ 1\ -\ i)$$
$$y\ =\ k_1 x/(k_2\ +\ x)$$
$$n\ =\ n\ +\ dt\ *\ y$$
$$z\ =\ k_3(n/volume)/(k_4\ +\ (n/volume))$$

Where i is the cell identity (1 or 0 for blue or yellow cells), x is the concentration of the cross-fed nutrient encountered by a cell. Nutrients (or signals) are named according to the cell that secreted them. $emit\_signal\ (\ i,\ 10\ )$ indicates that cell type i emits 10 units of nutrient type i at each time step. $get\_signal\ (\ 1\ -\ i)$ defines that a cell responds to the nutrient secreted by cells of the other type.

Chemotaxis

For panels a-c, the cells do not chemotax. For panel d, cells exhibit chemotaxis towards chemicals secreted by cells of the opposite type according to the chemotaxis program defined in Gro. For chemotaxis, the run parameter is set to 30 and the tumble parameter is set to 100.

Simulations shown in Figure 4

Parameter values

**Parameter**	**Value**
signal diffusion rate	0.1
signal degradation rate	0.01
maximum uptake rate, k_1	1
half saturation uptake constant, k_2	1
maximum growth rate, k_3	0.1
half saturation growth constant, k_4	1
time step of simulation, *dt*	.02 (default)
Initial positions blue cells, mixed pattern	(0,-20),(0,-20)

Initial positions blue cells, self pattern	(0,-20),(0,-20),(0,20),(0,20)
Initial positions yellow cells, mixed pattern	(0,20),(0,20)
Initial positions yellow cells, self pattern	(0,-20),(0,-20),(0,20),(0,20)

Growth equations

For all simulations, the cross-feeding growth equations (see above) were modified to reflect that amino-acids could either by supplied by the other cell type or from the external medium:

$$emit\_signal\,(\,i,\ 10\,)$$
$$x\ =\ get\_signal\,(\,1\ -\ i)$$
$$y\ =\ k_1(x\ +\ t)/(k_2\ +\ x\ +\ t)$$
$$n\ =\ n\ +\ dt\ *\ y$$
$$z\ =\ k_3(n/volume)/(k_4\ +\ (n/volume))$$

here t is the concentration of the amino acids in the external medium, and x the amount of amino-acids obtained from the partner cell type.

Chemotaxis

The yellow cells exhibit nutrient-dependent chemotaxis towards chemicals secreted by cells of the opposite type, and the blue cells do not chemotax. Chemotaxis is defined by Gro's built-in chemotaxis function, with run set to $t/40$ and tumble set to $t/5$.

Media nutrient concentration

The concentration of amino acid in the media, t, is varied, decreasing from left to right. The values of t used in the simulations are: 0.1, 1, 10, 100, 1000.

Supplementary References

M. Gutiérrez, P. Gregorio-Godoy, G. P. del Pulgar, L. E. Muñoz, S. Sáez, and A. Rodríguez-Patón. A New Improved and Extended Version of the Multicell Bacterial Simulator gro. ACS Synthetic Biology 2017 6 (8), 1496-1508
https://doi.org/10.1021/acssynbio.7b00003
